# Widespread Occurrence of Non-Extractable Fluorine in Artificial Turfs from Stockholm, Sweden

**DOI:** 10.1021/acs.estlett.2c00260

**Published:** 2022-07-06

**Authors:** Mélanie Z. Lauria, Ayman Naim, Merle Plassmann, Jenny Fäldt, Roxana Sühring, Jonathan P. Benskin

**Affiliations:** † Department of Environmental Science, Stockholm University, Svante Arrhenius Väg 8, 10691 Stockholm, Sweden; ‡ Department of Environment and Health, Nacka Municipality, Granitvägen 15, 131 81 Nacka, Sweden; § Department of Environment and Health, City of Stockholm, Fleminggatan 4, 104 20 Stockholm, Sweden; ∥ Department of Chemistry and Biology, Ryerson University, 350 Victoria Street, Toronto, ON M5B 2K3, Canada

**Keywords:** per- and polyfluoroalkyl substances, artificial turf, plastic, rubber, fluorine, fluoropolymers, processing aids, microplastic

## Abstract

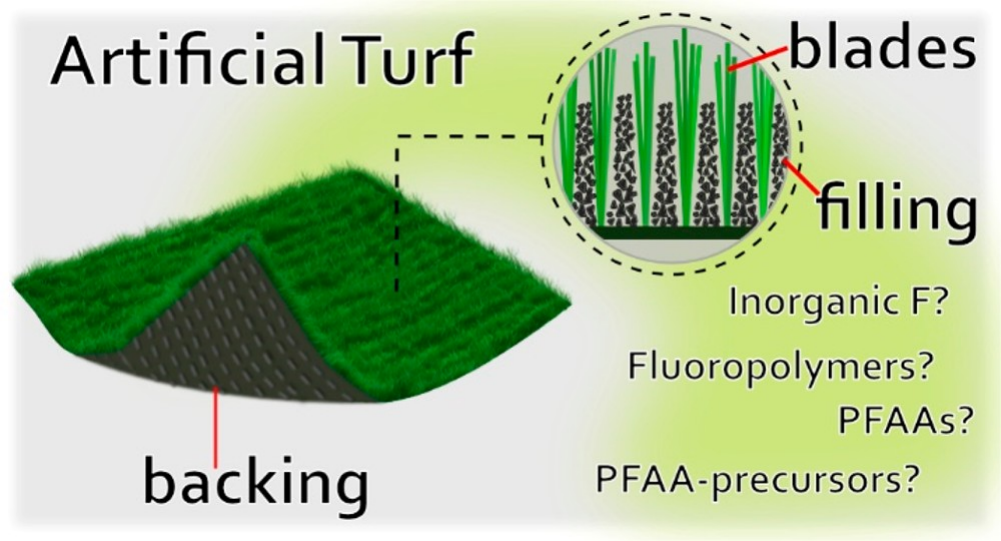

Per- and polyfluoroalkyl substances (PFAS) are frequently used in the production of rubber and plastic, but little is known about the identity, concentration, or prevalence of PFAS in these products. In this study, a representative sample of plastic- and rubber-containing artificial turf (AT) fields from Stockholm, Sweden, was subjected to total fluorine (TF), extractable organic fluorine (EOF), and target PFAS analysis. TF was observed in all 51 AT samples (ranges of 16–313, 12–310, and 24–661 μg of F/g in backing, filling, and blades, respectively), while EOF and target PFAS occurred in <42% of all samples (<200 and <1 ng of F/g, respectively). A subset of samples extracted with water confirmed the absence of fluoride. Moreover, application of the total oxidizable precursor assay revealed negligible perfluoroalkyl acid (PFAA) formation across all three sample types, indicating that the fluorinated substances in AT are not low-molecular weight PFAA precursors. Collectively, these results point toward polymeric organofluorine (e.g., fluoroelastomer, polytetrafluoroethylene, and polyvinylidene fluoride), consistent with patent literature. The combination of poor extractability and recalcitrance toward advanced oxidation suggests that the fluorine in AT does not pose an imminent risk to users. However, concerns surrounding the production and end of life of AT, as well as the contribution of filling and blades to environmental microplastic contamination, remain.

## Introduction

Per- and polyfluoroalkyl substances (PFAS) encompass a diverse group of >4700 chemicals defined as substances containing at least one fully fluorinated methyl or methylene carbon atom.^1^ When linked in series, fluorinated alkyl chains display a unique combination of hydrophobicity and lipophobicity, which makes them desirable across a wide range of industrial and consumer applications.^2^ While many PFAS have been linked with adverse health effects, the principle concern associated with all PFAS is their persistence.^3^ This property, combined with their widespread use, has led to the global distribution of PFAS in nearly all environmental compartments.^4−7^

Manufacturing of plastics and rubber is one of the major uses of PFAS (>4000 t between 2000 and 2017 in the Nordic countries alone, i.e., Sweden, Finland, Norway, and Denmark).^2^ Fluoropolymers are commonly added at low concentrations (100–1500 ppm) as polymer-processing aids (PPAs) to improve plastic extrusion.^8^ By reducing operating pressure, die build-up, and melt fracture, fluoropolymers increase the efficiency and quality of production. While fluoropolymer-based PPAs do not impart particular properties to the final plastic or rubber products, a low-level fluoropolymer residual may occur following production. In contrast, some plastics are intentionally modified with fluorine to enhance the performance of the final product.^9^ One example is direct perfluorination of polyolefins (e.g., high-density polyethylene), where hydrogen atoms on the outer surface of a material (top 10 μm) are replaced with fluorine. This treatment decreases the reactivity and permeability of the polymer, thereby broadening its applications. Direct perfluorination is known to produce low levels of perfluoroalkyl carboxylic acids (PFCAs) as reaction byproducts, which occur in the final product at low levels.^9^ The widespread use of PFAS in plastic manufacturing may explain the occurrence of these chemicals in plastic products such as artificial turf (AT).

AT is one of the more poorly understood uses of PFAS-containing plastic and rubber products. AT was first introduced as a replacement for natural grass in playgrounds and sports fields more than 50 years ago and comprise backing, blades, and filling.^10^ Backing usually consists of polyester or polypropylene with a secondary layer of latex or urethane,^11^ while blades are typically polyethylene and less frequently nylon or polypropylene.^12^ The filling materials are highly variable^12^ but have historically consisted mostly of crumb rubber made of shredded, recycled styrene–butadiene rubber (SBR) tires.^13,14^ Other fillings include virgin materials such as the ethylene propylene diene monomer (EPDM) and thermoplastic elastomers (TPE) or natural materials like sand, cork, coconut fiber, or walnut shells.^12,13^

Concerns surrounding AT have primarily centered around direct exposure of users to metals and (semi)volatile organic compounds contained in the crumb rubber filling (i.e., SBR).^15−17^ Leaching of contaminants and dispersion of blades and filling material/microplastics into stormwater also represent risks to the health of the surrounding environment.^18−20^ Beyond a single (non-peer-reviewed) U.S. study, which reported total fluorine (TF) and several individual PFAS in ATs,^21^ little is known about the prevalence or risks associated with PFAS in AT. The principle objective of this study was to investigate the occurrence and identity of PFAS in a representative sample of ATs in Stockholm, Sweden. Secondary objectives included investigating the relationship between PFAS concentrations and both field age and filling material. Given the number and diversity of PFAS known to exist, a wide range of measurements were applied to the turf, including determination of total and extractable organic fluorine (TF and EOF, respectively), targeted PFAS analysis, fluoride analysis, and the total oxidizable precursor assay (TOPA). Collectively, this work provides a comprehensive overview of fluorine in ATs and ultimately helps to improve our understanding of the importance of AT as a source of PFAS to the environment.

## Materials and Methods

### Sampling Design and Sample Collection

An inventory of 103 ATs was provided by the city of Stockholm (Stockholms Stad). Seven fields were excluded due to a lack of information or accessibility, leaving a total of 96 fields containing six different types of filling materials: thermoplastic olefins (TPO), TPE, SBR, sand, EPDM, and an organic filling (i.e., cork, bark, and coconut). Information about the blades and backing materials was not available. AT locations were mapped with Quantum Geographic Information System (QGIS), version 3.4-14-Madeira, and a stratified sampling method was applied to select two fields for each of the six filling types. In addition, the relationship between field age and PFAS concentrations was evaluated using eight fields (EPDM or TPE filling), four of which were newly installed after 2017 and four of which were installed prior to 2010. However, because the first stratum already included two old fields using TPE, the second stratum selected only six additional locations. In total, 18 locations were selected, but at the time of sampling, one field was turned into a temporary ice-skating rink, which reduced the final number of sampled fields to 17. Furthermore, in two fields (Hammarby IP and Knutby BP), sand was reported as the filling material, but an unidentified filling material was observed in addition to sand. These fields were marked as “unknown filling material” in the results. Further information about the sampling design and locations can be found in Table S1 and Figure S1.

Sample collection was conducted in February 2020. Gloves were used during sampling, and the equipment (scissors, knife, and spoon) was rinsed with methanol and air-dried prior to each sample collection. Blades of artificial grass were obtained by either cutting or collecting loose material from the field. Filling was collected from throughout the entire field. A cut of approximately 4 cm^2^ was made in each field to obtain the backing. All samples were stored in separate resealable plastic bags.

### Standards and Reagents

A total of 17 authentic and 13 isotopically labeled PFAS standards were used in this work, all of which were purchased from Wellington Laboratories (Guelph, ON). A full list of standards, including abbreviations, is provided in Table S2. Specific reagents used for sample preparation and analysis are also provided in the Supporting Information.

### Sample Extraction

Samples were not precleaned; TF measurements were performed directly on samples (i.e., no pretreatment), while EOF and targeted PFAS analyses were carried out following extraction using the following procedure.^22^ Samples were divided into three batches (blades, backing, and filling) with each batch including a method blank (empty tube) and quality control samples. Extractions were carried out with acetonitrile followed by an EnviCarb cleanup (see the Supporting Information for details). Thereafter, the extracts were split into clean Eppendorf tubes for combustion ion chromatography (CIC; 500 μL) and liquid chromatography tandem mass spectrometry analysis (LC-MS/MS; 250 μL). Since isotopically labeled PFAS contribute to the fluorine signal on the CIC, internal standards (ISTD; 1 ng) were only added after extraction to those extracts intended for LC-MS/MS analysis and extraction efficiencies were assessed with spike/recovery experiments (details in Quality Control). Finally, to assess the contribution of fluoride to TF measurements, a subset of samples were extracted using water and analyzed by CIC. Details of these extractions are provided in the Supporting Information, and the results are summarized in Table S3.

### Instrumental Analysis

Measurements of TF and EOF were carried out using a Thermo-Mitsubishi CIC using a previously developed method.^23^ Targeted PFAS analysis was performed on an Acquity ultraperformance liquid chromatograph (UPLC) using an Acquity UPLC BEH C18 column (2.1 mm × 50 mm, 1.7 μm particle size) coupled to a Xevo TQS tandem mass spectrometer (MS/MS) from Waters Corp. operated in negative electrospray ionization (ESI), multiple-reaction monitoring (MRM) mode, as described previously.^24^ Details of both CIC and LC-MS/MS methods are provided in the Supporting Information.

### Quality Control

The accuracy and precision of TF measurements were assessed through replicate combustions of certified reference material (CRM, BCR-461, fluorine in clay), which were included in each batch. Measurements showed good initial and ongoing agreement with the reference value (568 ± 60 mg of F/kg), with an average recovery of 91 ± 2%, indicating good method accuracy and precision.

Validation of EOF measurements was carried out using a series of spike/recovery experiments. First, samples of filling, blades, and backing were analyzed unfortified (*n* = 6 total), with a fortification of PFAS (278.62 ng of fluorine; *n* = 9 total), and with a fortification of inorganic fluorine (500 ng of NaF; *n* = 3 filling samples). The recovery for the PFAS mixture was 71 ± 19% (*n* = 9), indicating reasonable extraction and combustion efficiencies, while the recovery of NaF ranged from 1% to 3%, which showed good removal of inorganic fluorine during extraction. In addition, replicate (*n* = 9) analyses of BCR-461 during analysis of EOF extracts produced recoveries of 106 ± 3%, indicating the stability of the instrument during analysis.

The accuracy and precision of targeted PFAS analysis were assessed using replicate spike/recovery experiments with each of the three matrices (filling, backing, and blade). Spike/recovery experiments included both unfortified samples as well as samples spiked with 1 ng of individual PFAS. Blanks were also included, and all quality control samples were extracted in the same manner as real samples. Recoveries of target PFAS spiked into the organic filling (containing the lowest TF concentrations) ranged from 79% to 105% (1–11% RSD; *n* = 3), while the synthetic filling (highest TF concentrations) ranged from 73% to 134% (0.2–14% RSD; *n* = 2). For backing, recoveries ranged from 72% to 123% (2–13% RSD; *n* = 2) while for blades, recoveries ranged from 73% to 111% (3–22% RSD; *n* = 2) (see Figure S2 for details). The limit of detection (LOD) was used as the reporting limit and ranged from 3.40 to 198 pg/g (see Table S2).

### Total Oxidizable Precursor Assay

The TOPA is an oxidative conversion that facilitates indirect measurement of known and unknown PFAA precursors by converting them into easily measurable PFCAs.^25^ In the work presented here, a subset of samples with high TF was subjected to TOPA, including two blade samples, three backing samples, and four samples of filling. For quality control, the filling material with the lowest TF was also analyzed in triplicate with and without a fortification of 10 ng of *N*-ethyl-perfluoro-1-octanesulfonamidoacetic acid (EtFOSAA, average molar recovery of 59%). TOPA was carried out directly on the samples (as opposed to extracts), as described elsewhere^26^ and in the Supporting Information.

### Data Handling

To evaluate the fluorine mass balance, targeted PFAS concentrations (*C*_PFAS_; nanograms of PFAS per gram) were converted to their equivalent fluorine concentration (*C*_F_PFAS_; nanograms of F per gram) via the following equation:
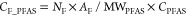
1where *N*_F_ is the number of fluorine atoms, *A*_F_ is the atomic weight of fluorine in grams per mole, and MW_PFAS_ is the molecular weight of an individual PFAS in grams per mole of PFAS.

### Inventory Calculations

The total quantity of fluorine from all ATs in Stockholm was estimated using the measured TF concentrations in backing, blades, and filling. The details of these calculations are provided in the Supporting Information. Briefly, for backing and blades, weight-based TF concentrations (i.e., micrograms of F per gram) were first converted to area-based concentrations (i.e., micrograms of F per square centimeter), which were thereafter multiplied by the area of the field to obtain the mass of TF per field for each of the respective components. For filling, weight-based concentrations (i.e., micrograms of F per gram) were multiplied by the estimated quantity of filling added to a field per year (2500 kg),^18^ with only one application considered in the calculation. Finally, the amounts of fluorine in backing, filling, and blades of a given field were summed to obtain the total amount of fluorine for that field. The largest and smallest quantities of measured TF were used as upper and lower bounds estimates, respectively, for the ATs not sampled (total of 86 sites). The sum of these estimates plus the measured values provided a total estimate of the quantity of fluorine in ATs in Stockholm.

## Results and Discussion

### Occurrence of PFAS and Fluorine in ATs from Stockholm

**Figure 1:**
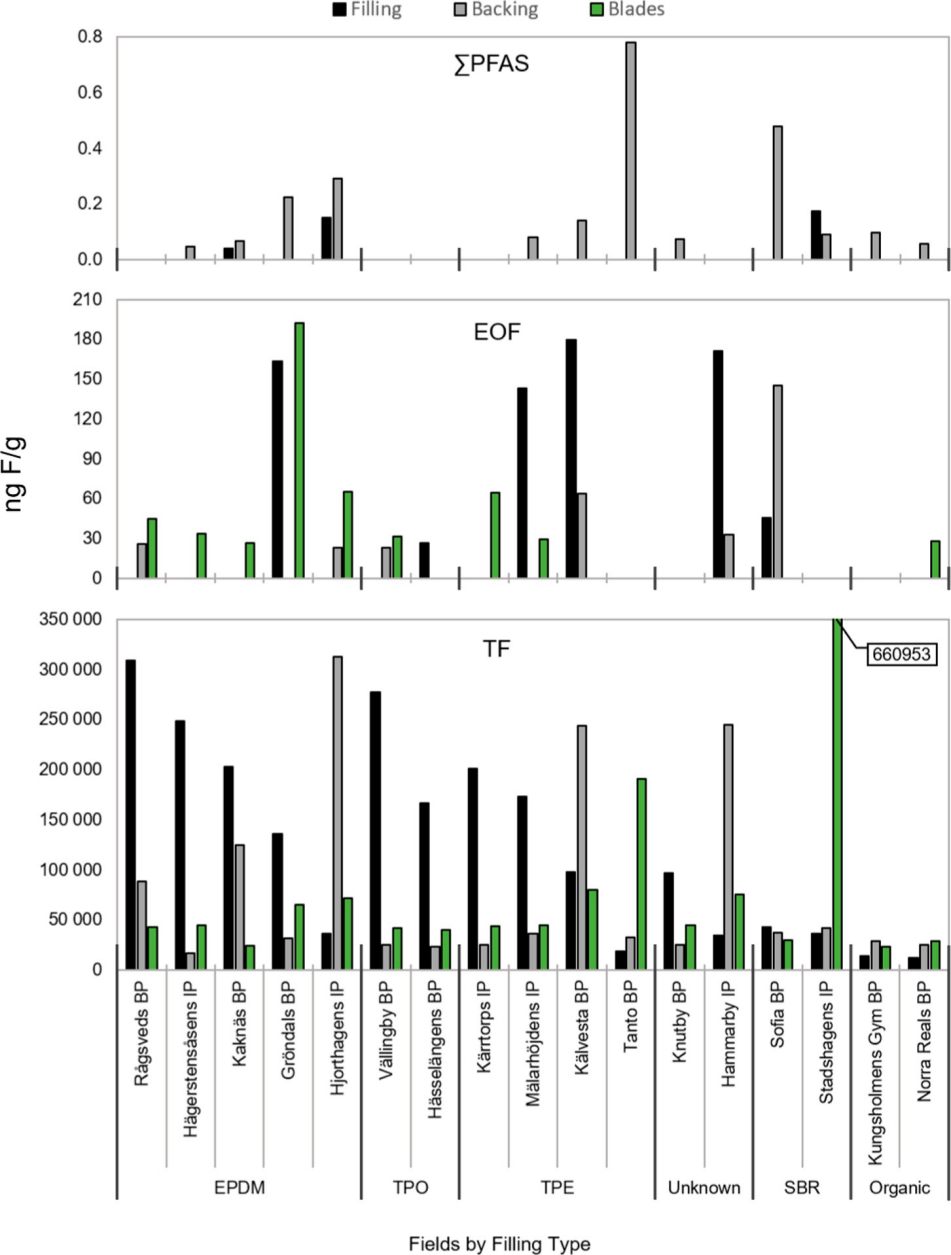
∑PFAS (top), EOF (middle), and TF (bottom) measurements grouped according to filling type and ordered according to decreasing TF concentrations.

CIC analysis revealed the presence of fluorine in all sample types from all locations in Stockholm (Figure 1 and Table S4), with TF concentrations ranging from 16 to 313 μg of F/g in backing, 12–310 μg of F/g in filling, and 24–661 μg of F/g in blades. These levels are similar to the range of concentrations reported previously in a non-peer-reviewed study of eight blade samples from the United States (44–255 μg of F/g).^21^ In contrast, EOF concentrations (Figure 1 and Table S4) were more than an order of magnitude lower than TF concentrations across all matrices, ranging from <LOD–145 ng of F/g (35% detection frequency [DF]) in backing, <LOD–179 ng of F/g (35% DF) in filling, and <LOD–192 ng of F/g (53% DF) in blades, indicating that most PFAS in AT materials are not extractable. To the best of our knowledge, this is the first time EOF has been determined in AT.

Target PFAS were detected intermittently and at low concentrations in backing (<LOD–0.63 ng of F/g; 71% DF) and filling (<LOD–0.15 ng of F/g; 18% DF) and were completely absent in blades (see Figure 1 and Table S4). Among target PFAS, long chain PFCAs (e.g., perfluorooctanoic acid, perfluorododecanoic acid, and perfluorotetradecanoic acid) were detected the most frequently and at the highest concentrations in backing, although reproducible patterns were not observed in the PFCA profile. In comparison, the U.S. report found PFOS in a discarded backing at 0.19 ng/g [0.12 ng of F/g (similar to this study)] and 6:2 fluorotelomer sulfonic acid in a new backing at 0.3 ng/g [0.17 ng of F/g (not included in this study)]. We speculate that the frequency of detection (in particular of long chain PFCAs) in backing from this work is higher because the underside is the least exposed to weathering and/or contributions from atmospheric deposition, and that long chain PFCAs are less mobile and more strongly sorbed to plastics compared to shorter chain-length homologues. However, differences in the occurrence and/or profile of PFAS may also arise from different types of plastics and ultimately their manufacturing process.

An investigation of the influence of filling material on measured concentrations revealed higher TF concentrations, albeit with considerable variability, in thermoplastics (TPO, 218.6 ± 77.9 μg of F/g; TPE, 119.7 ± 82.2 μg of F/g) and EPDM (183.3 ± 105.0 μg of F/g), compared to SBR (35.58 ± 4.91 μg of F/g) and organic material (9.58 ± 2.51 μg of F/g) (Figure 1). This finding was not observed among the EOF or target PFAS data. Furthermore, statistically significant differences between installation years (i.e., before 2010 vs after 2017) were not observed for TF, EOF, or target PFAS (two-sample *t* test; *p* > 0.05), and no relationship was observed among TF, EOF, and ∑target PFAS in any of the three AT components (Spearman’s correlation; *p* > 0.05).

### Disposition of Fluorine in AT

A series of additional experiments were performed to shed light on the identity of fluorine in the ATs. Application of the TOP assay to 10 samples (including backing, blades, and filling) containing high TF concentrations revealed negligible formation of PFCAs following oxidation (Figure S3). These results, combined with the low levels of PFAS and EOF, suggest that the fluorine in synthetic AT materials (i.e., not including organic fill) consists mostly of non-extractable, non-PFAA precursors, such as fluoropolymers. Contributions to TF arising from fluoride on the surface of AT materials were also ruled out on the basis of the negligible concentrations of fluorine in water extracts from samples of backing, blades, and synthetic filling compared to their high TF (see the Supporting Information and Table S3 for details). However, we cannot rule out contributions from inorganic fluorine species other than fluoride that may occur in the turf that could not be extracted in water. Ultra-short chain PFAS (possibly deposited via precipitation^27−29^) or non-PFAA-forming organofluorine residuals (such as those detected in other fluoropolymer-containing products^30^) may also contribute to the unidentified EOF observed in some samples. However, considering that EOF concentrations were typically more than 2 orders of magnitude lower than TF concentrations, substances making up the EOF appear to be relatively minor.

Collectively, these measurements point toward the occurrence of polymeric PFAS in AT components, which aligns with patent literature describing the use of polytetrafluoroethylene (PTFE) and fluoroelastomers as production processing aids and after treatment for polyethylene blades.^31,32^ PTFE and polyvinylidene fluoride (PVDF) are also mentioned in patents pertaining to AT filling where they are used as a coating treatment^33^ and a binding matrix.^34^ Unspecified organofluorine compounds are also added as fire retardants to filling material.^34^ While these examples are by no means a comprehensive list of all patents pertaining to PFAS in AT, they provide evidence that PFAS are used intentionally in AT production for a variety of reasons, in addition to plastic extrusion.

### Implications for Human and Environmental Exposure

While TF concentrations in ATs are considerable, the observation of poor extractability and recalcitrance toward advanced oxidation suggests that leaching and/or conversion to mobile PFAAs is limited over the lifetime of an AT and/or following accidental ingestion of AT components.^35^ However, further work is needed to investigate the fate of PFAS in plastics during weathering (e.g., by ultraviolet light), and caution is warranted when selecting plastic materials for use in AT to ensure they do not contain side chain fluorinated polymers (SFPs). While SFPs were not detected in ATs from this study (based on TOP results), they occur widely in plastics (in particular textiles) and may transform into PFAAs during weathering.^36^ Filling (and to some extent blades) also remains a highly problematic component of AT considering its potential to disperse into the environment as micro/nanoplastic (estimated between 1638 and 2456 t of filling in Sweden in 2016^18^), and considering the recent discovery of nanoplastics in human blood.^37^ Finally, concerns surrounding the production and end of life of AT remain. ATs analyzed in this study contained 0.315–17.439 kg of F per field (see Table S5 and Figure S4), which, when extrapolated to all fields in Stockholm, amounted to a sum total of 84.45–1557.16 kg of F that will eventually be landfilled or incinerated. Landfills are known sources of PFAS and microplastics,^38−40^ and the effectiveness of incineration for destroying PFAS remains unclear.^41−44^ The alternative, recycling of AT, is still a developing industry^45^ but will continue to be complicated by the use of PFAS and other additives in plastics,^46,47^ some of which will occur as impurities in recovered materials.^48^ Because manufacturers of the ATs investigated here are not exclusive to Sweden, we believe these results to be broadly translatable to ATs globally. Further research into the occurrence, stability, and environmental fate of PFAS in ATs, and plastics in general, is needed to better understand the implications of (re)use and disposal of AT components.

## Supporting Information Available

Details about sample preparation and analyses and details about artificial turf samples and inventory calculations (PDF)

## Notes

A preprint version of this work is available on *chemRxiv*.^49^
