# Supplementary material for: Widespread Occurrence of Non-Extractable Fluorine in Artificial Turfs from Stockholm, Sweden
Source: Environ Sci Technol Lett. 2022 Jul 6;9(8):666–72. doi: 10.1021/acs.estlett.2c00260 (PMC9367005; doi:10.1021/acs.estlett.2c00260)
Supplement: Supplementary file 1 — ez2c00260_si_001.pdf [file ez2c00260_si_001.pdf]

Widespread Occurrence of Non-Extractable Fluorine in Artificial Turfs from Stockholm, Sweden

Mélanie Z. Lauria,[1]* Ayman Naim,[1,2] Merle Plassmann,[1] Jenny Fäldt,[3] Roxana Sühring,[1,4#] and Jonathan P. Benskin[1]*[#]

[1]Department of Environmental Science, Stockholm University, Svante Arrhenius Väg 8, 10691, Stockholm, Sweden.

[2]Department of Environment and Health, Nacka Municipality, Granitvägen 15, 131 81, Nacka, Sweden.

[3]Department of Environment and Health, City of Stockholm, Fleminggatan 4, 104 20, Stockholm, Sweden.

[4]Department of Chemistry and Biology, Ryerson University, 350 Victoria St, Toronto, ON M5B 2K3, Canada.

*Corresponding authors:
Melanie.Lauria@aces.su.se
Jon.Benskin@aces.su.se

[#]RS and JPB contributed equally and share last authorship.

Extraction of samples

Approximately 1g of each sample was added to a 13 mL PP tube together with 7 mL of acetonitrile. Thereafter, the sample was vortexed and ultrasonicated for 15 minutes. After centrifugation (3500 rpm, 10 minutes), the supernatant was transferred to a new tube, and 6 mL of acetonitrile were added to the samples and the process was repeated. The extracts were combined and concentrated to ~1 mL under nitrogen, then transferred to 1.7 mL Eppendorf centrifuge tubes containing 25 mg of ENVI-Carb™ and 50 µL of glacial acetic acid for clean-up. Extracts were vortexed, centrifuged (10'000 rpm, 10 minutes) and split into clean Eppendorf tubes for CIC (500 µL) and LC-MS analysis (250 µL). Finally, 250 µL of 4 mM NH_4OAc in water and internal standards (ISTD, 1 ng) were added to the extracts for LC-MS analysis and all extracts were stored in the fridge until the day of analysis.

CIC Analysis

Prior to analysis, the sample carriers (ceramic boats) were cleaned by burning them at approximately 1100 °C. Each run started and finished with a calibration curve (100 µL of 50 µg/L to 25 mg/L of F^- solution, resulting in 5 to 2500 ng total injected) and a mid-level calibration standard was run repeatedly to monitor instrumental drift. Samples for TF analysis ranged from 0.01 to 0.1 g, while for EOF analysis 100 µL of extract were used. The mean fluoride concentration in the method blanks was subtracted from the samples in all cases. The limit of detection (LOD) for total fluorine (TF) analysis was defined as three times the standard deviation in the method blanks (388 ng/g). The LOD for extractable organic fluorine (EOF) analysis was defined in the same way and it was (22.4 ng/g).

LC-MS/MS Analysis

PFAS analysis was done on an Aquity ultra-performance liquid chromatograph coupled to a Xevo TQ-S tandem mass spectrometer (UPLC-MS/MS, Waters). Chromatographic separation was achieved injecting 5 µL onto an Acquity UPLC® BEH C18 Column (2.1 × 50 mm, 1.7 µm particle size) plus an Acquity UPLC BEH C18 VanGuard pre-column (2.1 × 5 mm, 1.7 µm particle size) from Waters. A mobile phase gradient using (A) 95% Milli-Q water and 5% acetonitrile with 2 mM ammonium acetate and (B) 95% acetonitrile and 5% Milli-Q water with 2 mM ammonium acetate started with 10% B,

increased to 100% B at 5 min, held constant for 3 min and equilibrated at 10% B for another 2 min. The electrospray ionisation source was operated in negative mode where the source and desolvation temperature were held at 150 °C and 350 °C, respectively, while the capillary voltage was set to 1 kV. Instrumental parameters were based on the method used in a previous study.[1] The monitored ion transitions for all PFAS can be found in Table S2. Quantification was performed using isotope dilution with an 8-point calibration curve (linear, 1/x weighting). Exactly matched, isotopically-labelled standards were used when available, otherwise a structurally similar isotopically-labelled standard was used (see Table S 3). The Limit of Detection (LOD) for individual PFAS was defined as three times the standard deviation of the blank for those PFAS appearing in blanks; for all others, the LOD was estimated as the concentration producing a signal-to-noise ratio above three, based on analysis of the lowest calibration standard. The LODs ranged from 3.4- 197.9 pg/g (Table S 3).

Total Oxidizable Precursor Assay Procedure

Approximately 40 mg of each sample were weighed in a 50 mL falcon tube to which were added 30 mL of MilliQ water, 0.48 g of potassium persulfate and 0.456 mL of NaOH (10 M). The tubes were placed in a temperature-controlled oven at 85ºC for 6 hours. After cooling samples were amended with 50 µl of a 200 pg/µl solution of isotopically labelled standards and their pH was adjusted between 5-9 using HCl (33%). Solid phase extraction (SPE) was carried out using Oasis WAX SPE cartridges (150 mg, 6 mL, Waters). Cartridges were conditioned with 4 mL each of 0.1% NH4OH in methanol, methanol and water after which samples were loaded and extracted under vacuum, followed by a rinse with 4 mL of MilliQ water. After drying, samples were eluted into new 13 mL tubes with 4 mL each of 0.1% NH4OH in methanol and methanol. Samples were dried to approximately 1 mL and 50 µl of a 200 pg/µl solution of isotopically labelled standards were added. An aliquot of 250 µL of sample was combined with 250 µL of 4 mM NH_4OAc in water for LC-MS/MS analysis.

[1] *Powley CR, George SW, Russell MH, Hoke RA, Buck RC. Polyfluorinated chemicals in a spatially and temporally integrated food web in the Western Arctic. Chemosphere. 2008;70(4):664-672.*

Inventory Calculations

The total quantity of fluorine from all artificial turfs (ATs) in Stockholm was estimated using the measured TF concentrations in backing, blades, and fill. Weight-based TF concentrations (i.e. µg F/g) in backing were first converted to area-based concentrations (i.e. µg F/cm^2) using a weight-to-surface-area conversion factor (1g/cm^2) which was approximately the same for all backing samples. The resulting concentrations were multiplied by the area of the field to obtain the mass of TF per field from backing. For fill, weight-based concentrations (i.e. µg F/g) were multiplied by the estimated quantity of fill added to a field per year (which is estimated between 2 and 3 metric tons, we therefore used 2500 kg for these calculations), only one application of fill for each field was considered in the estimation calculation.[2] For blades, weight-based concentrations (i.e. µg F/g) were converted to field area-based concentrations (µg F/cm^2) using a conversion factor (0.01g/blade) and the density of the blades on the turf (~10 blades/cm^2); thereafter, concentrations were multiplied by the area of the field to obtain the total quantity of fluorine from all of the blades in a single field. Finally, the amount of fluorine in backing, fill and blades of a given field were summed together to obtain the total amount of fluorine for that field. As an example calculation, Gröndals BP, which has a surface area of 4230 m^2, was found to have TF concentrations of 31984 ng F/g in backing:

$$F\left(Gr\ddot{o}ndals_{backing}\right) = 31\,984\ \frac{ng\ F}{g} \cdot 1\ \frac{g}{cm^2} \cdot 4\,230\ m^2\ =\ 1.35\ kg\ F$$

The TF concentration in filling was 136018 ng F/g:

$$F\left(Gr\ddot{o}ndals_{filling}\right)\ =\ 136\,018\ \frac{ng\ F}{g} \cdot 2500\ kg\ = 0.34\ kg\ F$$

And 31984 ng F/g in blades

$$F(Gr\ddot{o}ndals_{blades})\ =\ 64\,690\ \frac{ng\ F}{g} \cdot 100\,000\ \frac{blades}{m^2} \cdot\ 0.01\ \frac{g}{blade} \cdot 4\,230\ \ m^2 =\ 0.2736\ kg\ F$$

[2] *Magnusson K, Eliasson K, Fråne A, et al. IVL-report C 183 Swedish sources and pathways for microplastics to the marine environment: a review of existing data. Swedish Environmental Protection Agency. Published online 2017. Accessed April 14, 2022.*

The total amount of Fluorine for Gröndals is therefore estimated as:

$$F(Gröndals_{total}) = F(Gröndals_{backing}) + F(Gröndals_{filling}) + F(Gröndals_{blades})$$
$$= 1.35\ kg\ F + 0.34\ kg\ F + 0.2736\ kg = 1.967\ kg$$

Details of the TF measured for the other fields can be found in Table S5.

The sum of the total fluorine found in the sampled fields is 57.387 kg. The lowest amount of fluorine was found in Kungsholmens Gym BP at 0.315 kg, while the highest was found at Kälvesta BP at 17.439 kg. These values were used as upper and lower bounds estimates for the ATs not sampled and were multiplied by 86 (the remaining fields in Stockholm that were not sampled). The sum total of these estimates plus the measured values provided a total estimate of the quantity of fluorine in ATs in Stockholm, resulting in a range spanning from 84.45 kg to 1557.16 kg.

Extraction of Fluoride in Water

Contributions from fluoride towards TF measurements were ruled out based on the negligible concentrations of fluorine in water extracts from a subset of three samples of backing, blades, and filling containing high TF (backing from Hammarby IP, filling from Rågsveds BP and blades from Stadhagens IP). They were extracted twice with MilliQ water (7 and 6 mL) using an ultrasonic bath. The combined extracts were concentrated under nitrogen in a heated bath (60 °C) to ca 200 µL, which were then analysed by CIC. Results showed an average of less than 760 ng F/g, much lower than the TF values for these samples, while the same samples spiked with 5000 ng F^- showed recovery of fluoride in water extract of 65% (see Table S 3). However, we cannot rule out contributions from other inorganic fluorine species which may occur in the turf and were not extractable in water. Overall, these results point towards a polymeric organofluorine consistent with patent literature.

Table S 1 - Fields selected for sampling, Vasaparken was not sampled because the field had been turned into an ice-skating rink. In Hammarby IP and Knutby BP, sand was listed as the filling, but an unknown filling material was observed in addition to sand. The backing differed between fields as well, with some being thicker than others. In general, the new fields had thinner backing than the old fields but no inventory information was supplied regarding the nature of the backing material.

	**Field**	**Year of Installation**	**Filling Material**	**Blades Type**
1	VASAPARKEN	2008	EPDM	MONO
2	GRÖNDALS BP	2008	EPDM	MONO
3	RÅGSVEDS BP	2015	EPDM	MONO
4	HÄGERSTENSÅSENS BP	2015	EPDM	MONO
5	KAKNÄS BP	2019	EPDM	MONO
6	HJORTHAGENS IP	2019	EPDM	MONO
7	HAMMARBY IP	2012	Sand+Unknown	MONO
8	KNUTBY BP	2018	Sand+Unknown	MONO
9	STADSHAGENS IP B	2011	SBR	MONO
10	SOFIA BP	2013	SBR	MONO
11	MÄLARHÖJDENS IP	2008	TPE	MONO
12	KÄRRTORPS IP	2009	TPE	MONO
13	TANTO BP	2019	TPE	MONO
14	KÄLVESTA BP	2019	TPE	MONO
15	HÄSSELÄNGENS BP	2017	TPO	MONO
16	VÄLLINGBY BP	2017	TPO	MONO
17	KUNGSHOLMENS GYMN BP	2017	Organic Material	MONO
18	NORRA REALS BP	2017	Organic Material	Duo Shape

EPDM: ethylene propylene diene monomer; **SBR**: styrene butadiene rubber; **TPE**: thermoplastic elastomer; **TPO**: thermoplastic olefin; **MONO**: thick blades that stand upright; **Duo Shape**: a combination of MONO blades and slit-film blades, which are thinner blades obtained by cutting a thin sheet of plastic.

Table S 2 - PFAS targeted in this study (natives, internal standards and recovery standards) with MS parameters for quantification and limit of quantification, L-EtFOSAA was only targeted for analysis following total oxidizable precursor assay

Compound Name	Abbreviation	Precursor Ion	Quantitative Product Ion	Qualitative product ion	Internal Standard	IS transition	Native used for quantification	LOD (pg/g)
**Perfluorobutanoic acid**	**PFBA**	**213**	169		$^{13}C_4$-PFBA	217>172	PFBA	163
**Perfluoropentanoic acid**	**PFPeA**	**263**	219	169	$^{13}C_5$-PFPeA	266>223	PFPeA	81.0
**Perfluorohexanoic acid**	**PFHxA**	**313**	269	119	$^{13}C_2$-PFHxA	315>270	PFHxA	35.0
**Perfluoroheptanoic acid**	**PFHpA**	**363**	319	169	$^{13}C_4$-PFHpA	367>322	PFHpA	43.0
**Perfluorooctanoic acid (linear)**	**L-PFOA**	**413**	169	369	$^{13}C_4$-PFOA	417>372	L-PFOA	43.0
**Perfluorooctanoic acid (branched)**	**Br-PFOA**	**413**	169	369	$^{13}C_4$-PFOA	417>372	L-PFOA	43.0
**Perfluorononanoic acid**	**PFNA**	**463**	419	219	$^{13}C_5$-PFNA	468>423	PFNA	197.9
**Perfluorodecanoic acid**	**PFDA**	**513**	469	269	$^{13}C_2$-PFDA	515>470	PFDA	79.5
**Perfluoroundecanoic acid**	**PFUnDA**	**563**	519	269	$^{13}C_2$-PFUnDA	565>520	PFUnDA	140.9
**Perfluorododecanoic acid**	**PFDoDA**	**613**	569	169	$^{13}C_2$-PFDoDA	616>570	PFDoDA	54.0
**Perfluorotridecanoic acid**	**PFTriDA**	**663**	619	169	$^{13}C_2$-PFDoDA	616>570	PFTriDA	135.9
**Perfluorotetradecanoic acid**	**PFTeDA**	**713**	669	169	$^{13}C_2$-PFDoDA	616>570	PFTeDA	51.0
**Perfluoropentadecanoic acid**	**PFPeDA**	**763**	719	169	$^{13}C_2$-PFDoDA	616>570	PFTeDA	51.0
**Perfluorobutanesulfonic acid**	**PFBS**	**299**	80	99	$^{18}O_2$-PFHxS	403>84	PFBS	53.0
**Perfluorohexanesulfonic acid (linear)**	**L-PFHxS**	**399**	80	99	$^{18}O_2$-PFHxS	403>84	L-PFHxS	40.0
**Perfluorohexanesulfonic acid (branched)**	**B-PFHxS**	**399**	80	99	$^{18}O_2$-PFHxS	403>84	L-PFHxS	40.0
**Perfluorooctanesulfonic acid (linear)**	**L-PFOS**	**499**	80	99	$^{13}C_4$-PFOS	503>80	L-PFOS	137.0
**Perfluorooctanesulfonic acid (branched)**	**B-PFOS**	**499**	80	99	$^{13}C_4$-PFOS	503>80	L-PFOS	43.0
**Perfluorodecanesulfonic acid (linear)**	**L-PFDS**	**599**	80	99	$^{13}C_4$-PFOS	503>80	L-PFDS	46.0
**Perfluorodecanesulfonic acid (branched)**	**B-PFDS**	**599**	80	99	$^{13}C_4$-PFOS	503>80	L-PFDS	46.0
**Perfluorooctanesulfonamide (linear)**	**L-PFOSA**	**498**	78	478	$^{13}C_8$-PFOSA	506>78	L-PFOSA	42.0
**Perfluorooctanesulfonamide (branched)**	**B-PFOSA**	**498**	78	478	$^{13}C_8$-PFOSA	503>80	L-PFOSA	42.0
**N-Ethyl perfluorooctanesulfonamidoacetic acid**	**L-EtFOSAA**	**584**	419	526	D_5-EtFOSAA	589>419	L-ETFOSAA	3.40

Table S 3 – Details of extraction of fluoride in water and TF concentrations (ng F/g) for comparison.

Sample	Spike (ng)	Measured F (ng; blank substracted)	Concentration (ng F/g)	Recovery of F (%)	TF (ng F/g)
**Hammarby IP (backing)**		<LOD	<LOD		244831
**Rågsveds BP (filling)**		3.21	37.32		309531
**Stadhagens IP (blades)**		90.53	2240.77		660953
**Hammarby IP (backing) + F**	5000	2828.19	82777.22	58.45	
**Rågsveds BP (filling) + F**	5000	3416.75	196402.2	68.35	
**Stadhagens IP (blades) + F**	5000	3385.59	40821.35	65.84	

LOD: limit of detection

Table S 4 – Results of TF, EOF analysis and ∑PFAS in F equivalents

Field	Backing			Filling			Blades		
	**PFAS ng F/g**	**EOF ng/g**	**TF ng/g**	**PFAS ng F/g**	**EOF ng/g**	**TF ng/g**	**PFAS ng F/g**	**EOF ng/g**	**TF ng/g**
Gröndals BP	0.225	<LOD	31984	<LOD	163.2	136018	<LOD	192.1	64690
Hammarby IP	<LOD	32.8	244831	<LOD	171.1	34421	<LOD	<LOD	75249
Hjorthagens IP	0.293	23.3	312986	0.150	<LOD	36210	<LOD	65.3	71406
Hägerstensåsens IP	0.047	<LOD	16480	<LOD	<LOD	248792	<LOD	33.6	44670
Hässelängens BP	<LOD	<LOD	23437	<LOD	26.2	166842	<LOD	<LOD	39827
Kaknäs BP	0.066	<LOD	124370	0.039	<LOD	203309	<LOD	26.2	24284
Knutby BP	0.074	<LOD	24713	<LOD	<LOD	96913	<LOD	<LOD	44617
Kungsholmens Gym BP	0.095	<LOD	28648	<LOD	<LOD	14238	<LOD	<LOD	23564
Kälvesta BP	0.140	63.6	243914	<LOD	179.2	97490	<LOD	<LOD	80341
Kärrtorps IP	<LOD	<LOD	24778	<LOD	<LOD	201294	<LOD	64.7	43840
Mälarhöjdens IP	0.079	<LOD	36022	<LOD	143.3	173539	<LOD	29.4	44367
Norra Reals BP	0.056	<LOD	25210	<LOD	<LOD	11909	<LOD	27.8	29073
Rågsveds BP	<LOD	25.7	88310	<LOD	<LOD	309531	<LOD	44.8	43088
Sofia BP	0.480	144.9	37159	<LOD	45.2	43115	<LOD	<LOD	29356
Stadshagens IP	0.089	<LOD	42268	0.173	<LOD	36183	<LOD	<LOD	660953
Tanto BP	0.782	<LOD	32276	<LOD	<LOD	18439	<LOD	<LOD	190717
Vällingby BP	<LOD	22.9	25048	<LOD	<LOD	277112	<LOD	31.1	41818

Table S 5 – Details of calculations estimating the total amount of Fluorine present in the sampled fields

		Backing			Blades				Filling		Total
**Field**	**Size (m^2)**	**TF (ng F/g)**	**TF (ng F/cm^2)**	**F (kg)**	**TF (ng F/g)**	**Number of blades**	**Weight of Blades (g)**	**F (kg)**	**TF (ng F/g)**	**F (kg)**	**F in Field (kg)**
Gröndals BP	4230	31984	31984	1.353	64690	4.23E+08	4.23E+06	0.2736	136018	0.3400	1.967
Hammarby IP	540	244831	244831	1.322	75249	5.40E+07	5.40E+05	0.0406	34421	0.0861	1.449
Hjorthagens IP	800	312986	312986	2.504	71406	8.00E+07	8.00E+05	0.0571	36210	0.0905	2.652
Hägerstensåsens IP	2400	16480	16480	0.396	44670	2.40E+08	2.40E+06	0.1072	248792	0.6220	1.125
Hässelängens BP	2400	23437	23437	0.562	39827	2.40E+08	2.40E+06	0.0956	166842	0.4171	1.075
Kaknäs BP	7140	124370	124370	8.880	24284	7.14E+08	7.14E+06	0.1734	203309	0.5083	9.562
Knutby BP	6000	24713	24713	1.483	44617	6.00E+08	6.00E+06	0.2677	96913	0.2423	1.993
Kungsholmens Gym BP	900	28648	28648	0.258	23564	9.00E+07	9.00E+05	0.0212	14238	0.0356	0.315
Kälvesta BP	6825	243914	243914	16.647	80341	6.83E+08	6.83E+06	0.5483	97490	0.2437	17.439
Kärrtorps IP	6825	24778	24778	1.691	43840	6.83E+08	6.83E+06	0.2992	201294	0.5032	2.494
Mälarhöjdens IP	6825	36022	36022	2.458	44367	6.83E+08	6.83E+06	0.3028	173539	0.4338	3.195
Norra Reals BP	1890	25210	25210	0.476	29073	1.89E+08	1.89E+06	0.0549	11909	0.0298	0.561
Rågsveds BP	2400	88310	88310	2.119	43088	2.40E+08	2.40E+06	0.1034	309531	0.7738	2.997
Sofia BP	2400	37159	37159	0.892	29356	2.40E+08	2.40E+06	0.0705	43115	0.1078	1.070
Stadshagens IP	6000	42268	42268	2.536	660953	6.00E+08	6.00E+06	3.9657	36183	0.0905	6.592
Tanto BP	800	32276	32276	0.258	190717	8.00E+07	8.00E+05	0.1526	18439	0.0461	0.457
Vällingby BP	6000	25048	25048	1.503	41818	6.00E+08	6.00E+06	0.2509	277112	0.6928	2.447

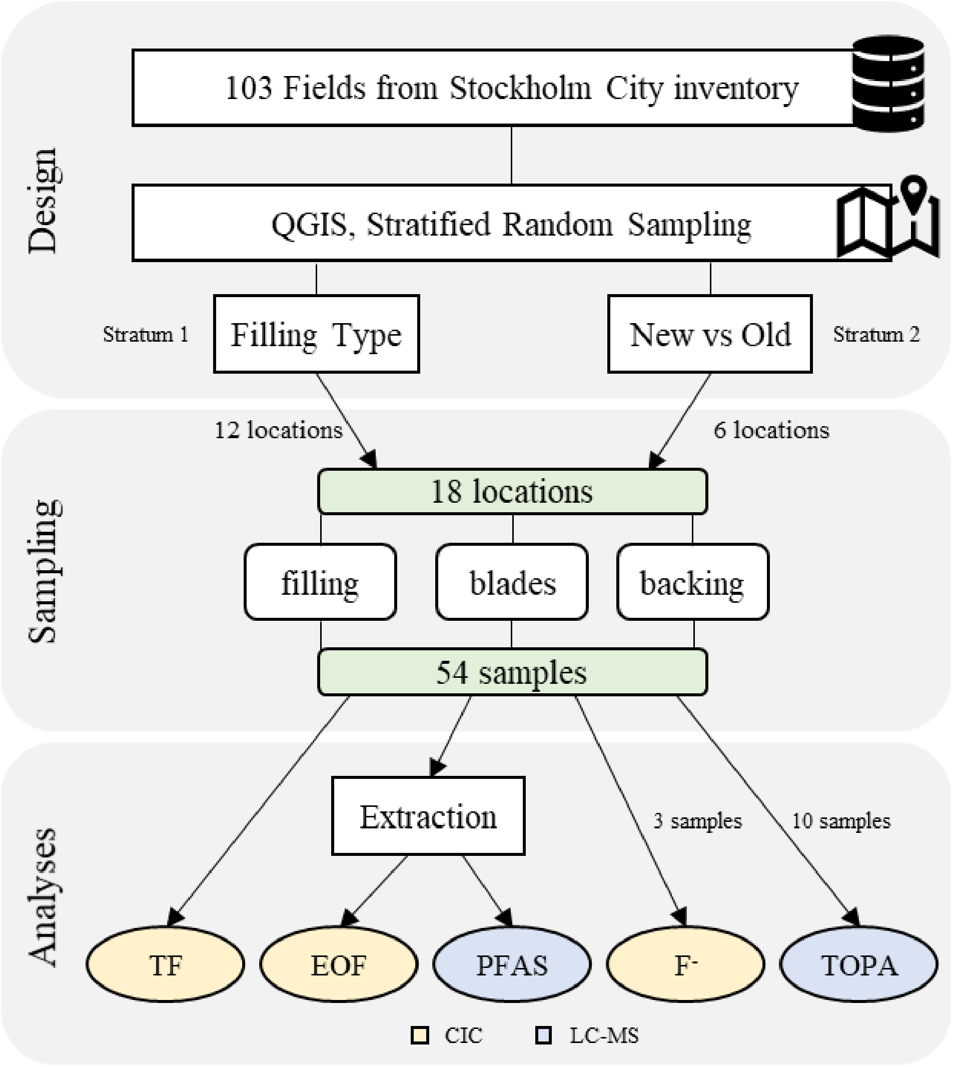

Figure S 1 - Overview of experimental approach. A stratified random sampling design was applied using two strata. In the first stratum, 2 fields for each filling type were selected while in the second stratum, 2 fields installed pre-2010 and 2 fields installed after 2017 were selected for filling types EPDM and TPE. For each location, samples of backing, blades and filling were collected. All samples were subjected to TF, EOF and targeted PFAS analyses. Smaller subsamples were selected for F^- and TOPA analyses.

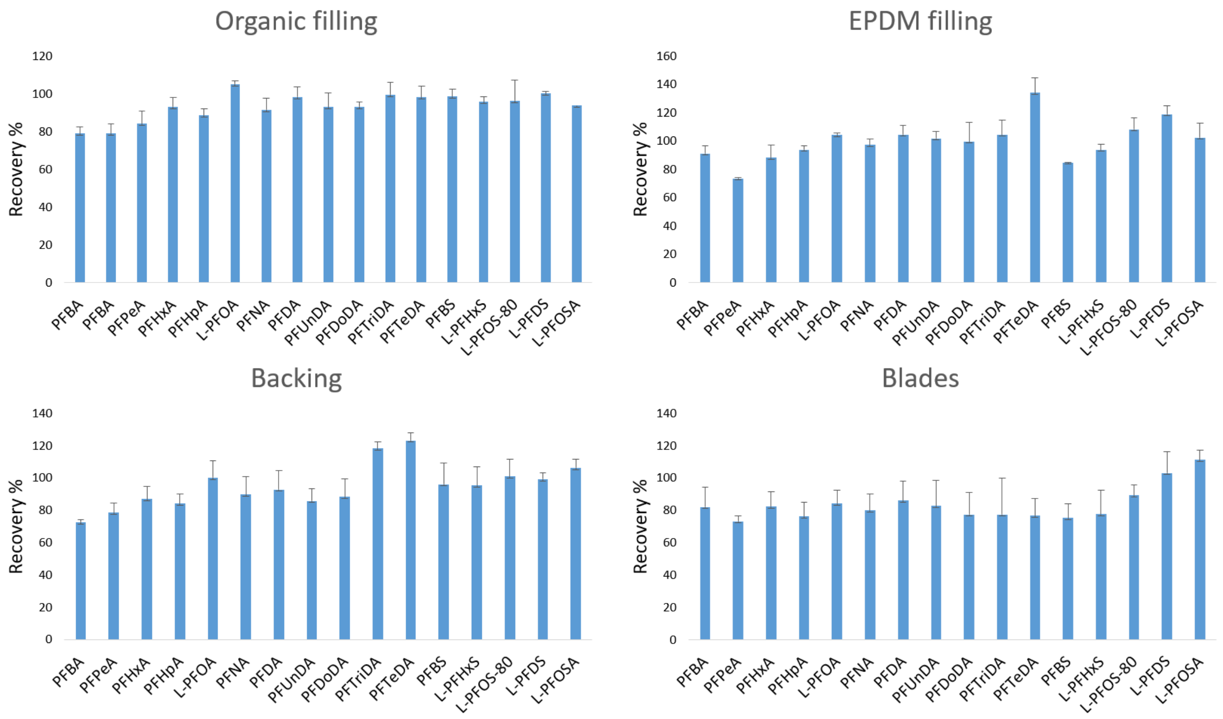

Figure S 2 - Percent recovery ± RSD of native PFAS spiked onto two types of filling: organic material (n=3 spiked samples) and EPDM (n=2 spiked samples), as well as backing (n=2 spiked samples), and blades (n=2 spiked samples

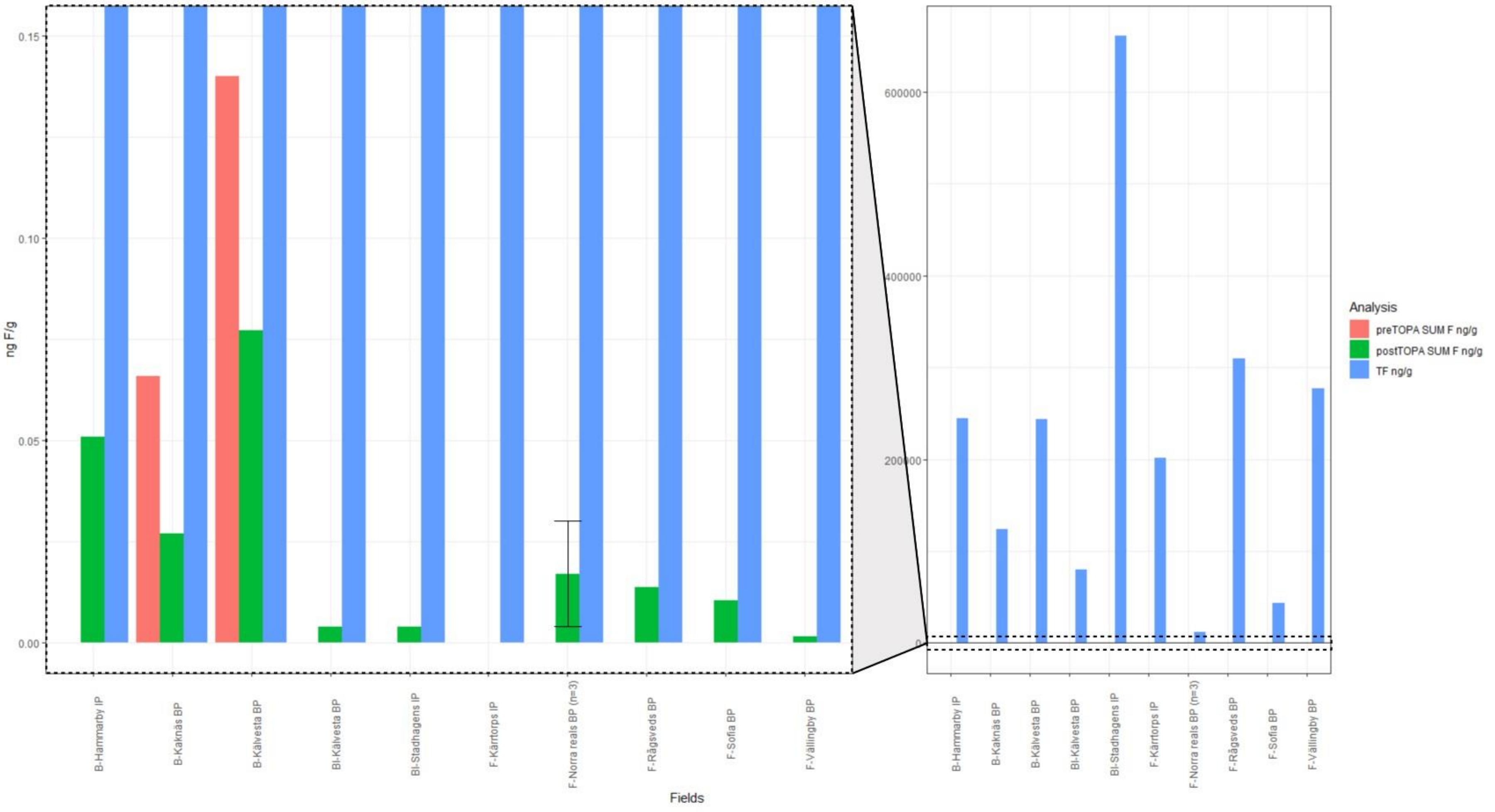

Figure S 3 - Results of TOPA performed on samples of backing (B), Blades (Bl) and filling (F). The lower ∑PFAS concentration after TOPA for some samples can be explained by more losses in the more complicated sample preparation for TOPA analysis.

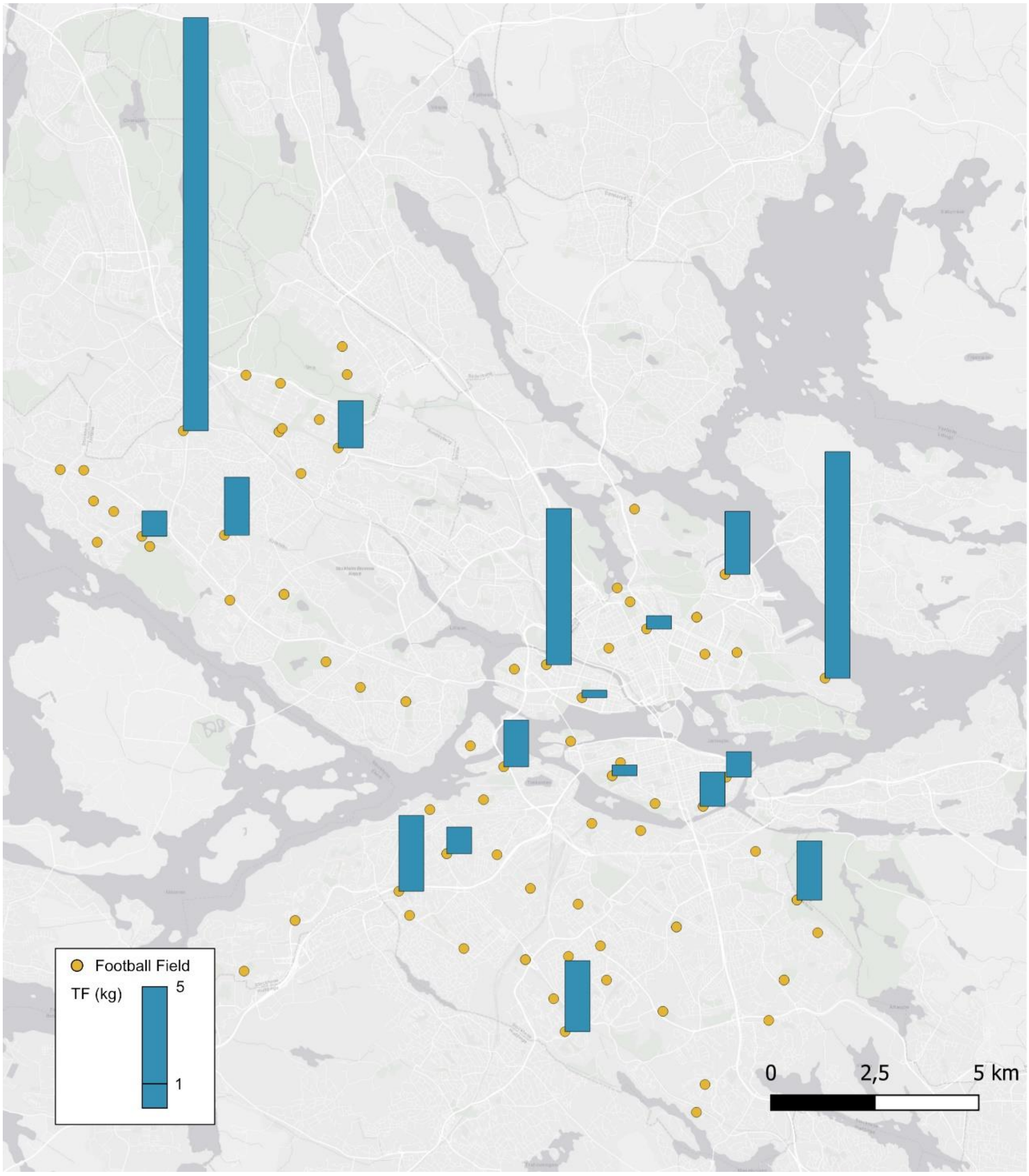

Figure S 4 – Artificial Turf Fields in Stockholm, TF amounts of the 17 fields investigated are shown.
